# Open-source hardware for medical devices

**DOI:** 10.1136/bmjinnov-2015-000080

**Published:** 2016-03-14

**Authors:** Gerrit Niezen, Parisa Eslambolchilar, Harold Thimbleby

**Affiliations:** Department of Computer Science, Swansea University, Swansea, UK

**Keywords:** Affordable, Inventions, Global Health

## Abstract

Open-source hardware is hardware whose design is made publicly available so anyone can study, modify, distribute, make and sell the design or the hardware based on that design. Some open-source hardware projects can potentially be used as active medical devices. The open-source approach offers a unique combination of advantages, including reducing costs and faster innovation. This article compares 10 of open-source healthcare projects in terms of how easy it is to obtain the required components and build the device.

## Introduction

A wide range of open-source hardware that includes healthcare sensors and low-cost single-board computers are readily available on the consumer market. The Arduino, arguably one of the most widespread and successful open-source hardware platforms, contains a microcontroller with an easy-to-use development environment that makes it ideal to build devices. It has a large community of supporters, and there are open software libraries available to simplify many tasks.

The radical shift in approach is that these new designs are *open*. In principle, anybody can build an Arduino, because its full design specification is available for anybody to use. This openness has stimulated many people to build a huge range of sensors and other devices that are compatible with it. Ironically, being open rather than protecting their designs (eg, with patents) has vastly increased their sales and lowered prices through stimulating a worldwide marketplace.

Why is open-source hardware a good idea for medical devices? Making the hardware design available under an open-source license allows anyone to improve and contribute to the device design, leading to very rapid innovation compared to traditional methods.^1^ ^2^ It also enables the design to be modified for very specific uses, and makes the devices easy to repair, factors which also reduce the impact these devices have on the environment. An open-source medical device increases safety, security and robustness by allowing more people to inspect and improve its designs.^3^ In fact, it is so difficult to develop good computer security that the best encryption algorithms are always developed in the open to allow others to inspect and improve them.^4^ Furthermore, if the software to connect devices is open-source and the physical interfaces standardised, vendor lock-in can be prevented. Open-source also allows many manufacturers to use the same design but differentiate in other ways in order to compete, for example on usability, support or wider interoperability with other devices. The same approach can improve medical devices.

### Potential of open-source medical devices for the developing world

According to the WHO, 70–90% of all medical devices donated to the developing world never function as intended.^5^ ^6^ Very simple faults, like a broken fuse or dead batteries, account for 15% of these failures. Twenty per cent of all donated equipment are not used because there are no manuals available or because of poor user training. Even when training is provided, it is rare for technical staff to be provided with technical training.

How would an open-source medical device like a syringe pump solve some of these problems? A new syringe pump currently on the market can cost between $500 and $10k, depending on the number of features and its application. Open-source syringe pumps can be built for 5–10% of the cost of a pump of similar performance, which has great potential for making medical devices more accessible in the developing world,^7^ where devices can also be designed as open-source and built for specific use cases, instead of having to depend on donated equipment from first world countries.

Arguably, under-resourced ‘developed’ healthcare has exactly the same problems though on a different scale: open-source should therefore have similar benefits in the developed healthcare systems.

### Quantifying the value of open-source hardware development

Pearce^1^ developed a set of formulas to quantify the value of open-source hardware design. One way is to compare distributed manufacturing to traditional manufacturing, based on the number of downloads of a design that results in a manufactured product. Another way is to calculate the costs saved by not having to replicate a product design, based on the number of design hours and hourly wage of the designers. A third approach is to calculate the market size of a distributed manufacturing approach based on the number of products and their manufacturing cost.

Using the first approach above, the value of a basic syringe pump design by Wijnen *et al*^8^ was shown to be between $778 000 and $12.4 million over 1 year. This is called the *downloaded substitution valuation*, which calculates the annual savings by comparing the cost of purchasing a traditionally manufactured product to the marginal cost of producing the open-source hardware version using distributed manufacturing. The value of the design is further quantified using the number of times that the open-source hardware design is downloaded.

### Regulatory issues

Clinical trials for medical devices may take anything from 2 to 5 years. While 5 years is a long time for a small business, performing the design and development, and the clinical trials, as part of an open-source project means that a small business can share the load. In principle the business would need to be in operation for a much shorter time before it could successfully launch a new product.

## Methods

We selected 10 open-source hardware medical devices and compared them in terms of how easy it is to obtain the components and build the device independently. A systematic review is not possible at this stage, as many of the projects are still in early phases and are not yet reported in peer-reviewed literature. As this is a rapidly developing area, then, we do not claim our list of projects is exhaustive or fully representative. We defined the scope of the review based on the following inclusion criteria: (1) availability of source files, and (2) can potentially be used as an *active* medical device, that is, it relies on software and a source of electrical energy for functioning.

All the design files, for example, two-dimensional drawings, CAD (computer-aided design) files, circuit schematics and layouts, need to be available and modifiable. If the files are only available in a non-editable format, the project was excluded. For example, the Robohand^9^ three-dimensional (3D)-printed prosthetics project does not share source files, only STL (STereoLithography) files, and then only in a non-editable format.

We distinguish between:

- Passive medical devices, like hose clamps
- Medical devices relying on software, like most syringe drivers
- Devices for laboratory research, like centrifuges

## Project comparison

### Myoelectric prostheses

Myoelectric prostheses are controlled by tiny voltages generated on the surface of the skin by the activity of residual muscles, called surface electromyography (EMG). These voltages are amplified and processed so that when the user flexes a muscle, motors on the prosthesis move in a predictable way. A myoelectric prosthesis does away with the harness and cables of a body-powered device and can be made to look very natural, although at the cost of a more restrictive and less comfortable socket. Another downside to these devices is their lack of responsiveness: most prosthetic hands move slowly to increase grip force or battery life at the expense of fast action. However, the major barrier to adoption is cost: an arm costs at least $30 000.

#### MyOpen

MyOpen,^10^ part of the Open Prosthetics Project,^11^ creates open-source hardware for myoelectric control. A circuit board design for a digital signal processing unit and related software are available.^12^ The hope is that it can eventually be used to control mechatronic prostheses for amputees.^13^ MyOpen wants to bring the cost of a prosthetic arm as low as $250.

Silva *et al*^14^ have made and tested a self-contained mechanomyogram controlled prosthesis. Their device contains three sensors, each consisting of a microphone to pick up muscle sounds and an accelerometer to detect external interference. Their signals are interpreted by a microcontroller and converted into simulated EMG signals to control an Otto Bock hand.^15^ Their results show that the devices perform with over 70% control accuracy.

In an attempt to replicate the research conducted by Silva *et al*, the Open Prosthetics project^11^ built a data collection system for recording MMG signals. It consists of a 3.5 mm audio plug, a microphone cartridge, some wire and a laptop. Not including the laptop, the components cost less than $5. The microphone is an electret condenser type, and is powered by the laptop's sound card. They have used the free and open-source software (FOSS) Audacity to record and process the sound. They have taped the microphone directly onto the skin with masking tape, plugged it into the laptop, and recorded the audio. No special skills or tools are required to build this system.

#### e-Nable

Another effort to develop a myoelectric prosthetic is the Limbitless Arm,^16^ created by a team at University of Central Florida. This prosthetic is also part of e-Nable,^17^ an online community that connects amputees with people who have 3D printers. It was estimated to cost approximately $350 in materials to produce a Limbitless Arm.

To produce a 3D printed mechanical hand, 3DUniverse^18^ is selling a ‘Hand Materials Kit’ for $25. This kit is for assembling the printed hand only and is also developed by the e-Nable community. The models are available to download freely and print.^17^ However, the users still need to access a 3D printer to print the parts. Note that inexpensive 3D printers are available in developing world contexts with some open-source printers even designed to be mobile.^19^

### CT scanner

**Figure 1:**
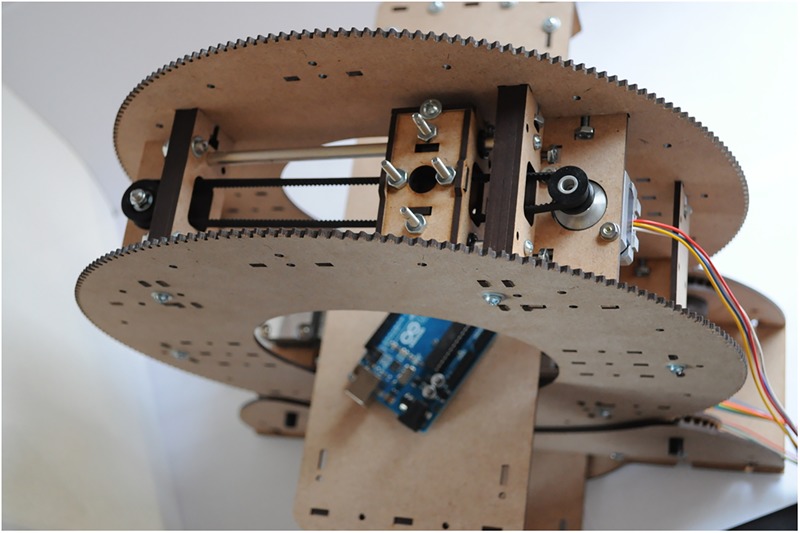
Open-source CT scanner (Photo by Peter Jansen, image licensed under the Creative Commons BY-SA license).

Jansen^20^ built an open-source desktop CT scanner (see figure 1) for small objects. The complete design files are available online,^21^ including the mechanical design, circuit schematics and firmware. The CT scanner uses a very low intensity radioisotope X-ray source, which means that even low resolution images take hours of measurement.^22^ It is designed for academic and educational purposes, but the hope is that it could eventually be used as a medical scanner in developing countries.

Jansen designed the almost entirely laser-cuttable CT scanner with four axes of motion, one being a large rotary gantry. The stepper motor to rotate the gantry is a National Electrical Manufacturers Association (NEMA)17 stepper from open-source hardware distributor Adafruit ($14), which transfers motion to the drive shaft using a belt and timing pulleys. Each linear axis has a small carriage that contains mounts for either the source or detector. Each axis has an inexpensive NEMA14 stepper also from Adafruit (under $14) and an idler pulley.^23^ Jansen has used a very small solid state high-energy particle detector called the type-5 from radiation watch, which can be connected to an external microcontroller. The price of this particle detector is just under $80.

To test out the motion and detector, Jansen put together an Arduino shield ($45) with three Pololu stepper controllers^24^ and a connector for the detector. An SD card slot can store the image data for large scans.

The source of radiation used in Jansen's design is Barium-133 (cost between $80 and $125.00) with 80–383 KeV energy. This radioisotope check source is sealed in epoxy, and is of such low intensity that it not licensed^25^ and considered safe unless the material is digested or taped to the body for long periods, and it can be disposed of as general trash. The total cost of Jansen's open-source CT scanner (excluding the laser cutter) is about $300. Open-source laser cutters can be built for around $1000. All required components are readily available and only basic knowledge of electronics and laser cutting is required.

### Infusion pumps

Infusion pumps dispense fluid over a set amount of time. In hospitals they are used to deliver medication for treatments like chemotherapy and pain management, while laboratories use them for everything from microfluidics to bioprinting.

#### Fechko's peristaltic pump

**Figure 2:**
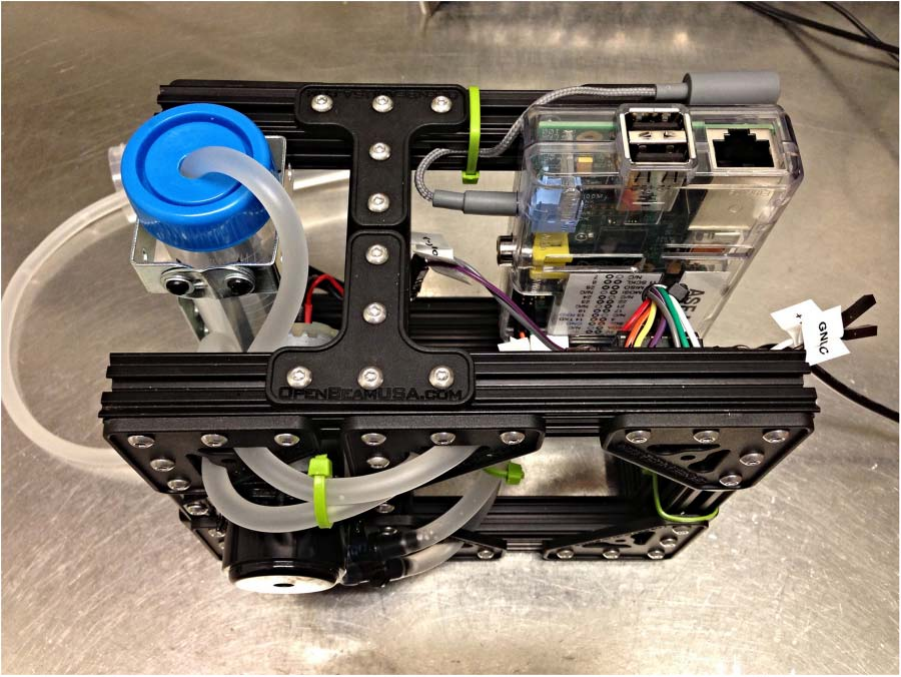
Raspberry Pi Peristaltic Pump (Photo by Amber Fechko, image licensed under the Creative Commons BY-SA license).

Fechko^26^ built a peristaltic pump (see figure 2) using a Raspberry Pi single-board computer and a standardised T-slot profile construction set called OpenBeam. The pump was designed as a fluid reward system for laboratory animals, but can be adapted for other uses as well.

The electronics consist of modular components that do not require soldering. The electronics used are an Arduino or Raspberry Pi and components easily obtained from open-source hardware distributors. Source code is available for Arduino, using a motor shield, and Raspberry Pi, using either a Gertboard or L293D motor control chip. All components used are available off-the-shelf and no special skills are required to build the pump.

#### Wijnen *et al*'s syringe pump

Wijnen *et al*^8^ published a complete open-source syringe pump library, consisting of a series of 3D-printable parts to build a syringe pump. Performance of the syringe pump was assessed and the methods used for assessment are detailed. The design, bill of materials and assembly instructions are publicly available. The 3D-printable parts can be modified using an open-source parametric 3D design tool, called OpenSCAD^27^ and derivatives like OpenPump^28^ ^29^ have already been created.

To build the device a 3D printer, or 3D printing service, can be used to print the necessary parts. The electronics are based on a Raspberry Pi, stepper motor and driver components that can be obtained from open-source hardware distributors. Most mechanical components can be obtained from a local hardware store. Some specialist components like ball bearings, linear bearings and flexible couplings can be sourced online. Source code is available for the Raspberry Pi.

### Physiological monitoring

#### e-Health sensor platform

**Figure 3:**
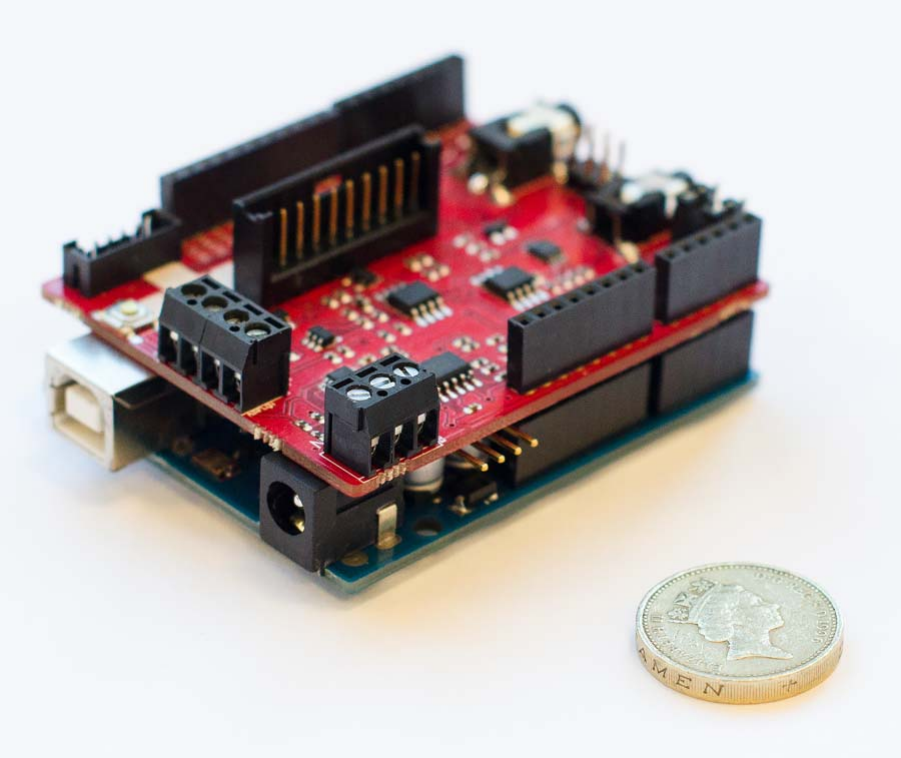
Arduino (bottom, showing USB connector) and e-Health shield. UK £1 coin (2.25 cm diameter) for scale. (Photo by Patrick Oladimeji, image licensed under the Creative Commons BY-SA license).

**Figure 4:**
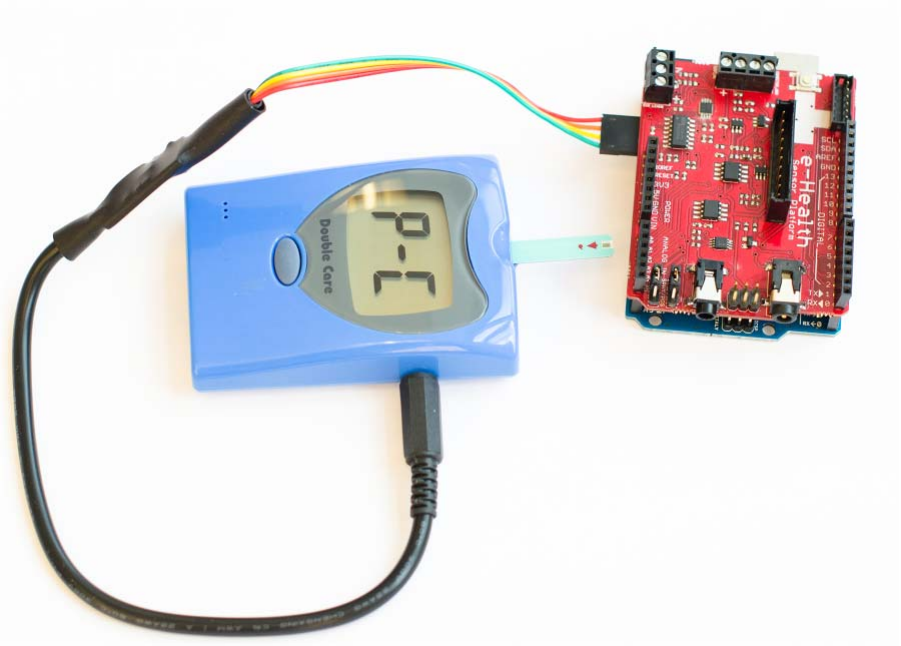
Blood glucose meter connected to e-Health shield (Photo by Patrick Oladimeji, image licensed under the Creative Commons BY-SA license).

The e-Health Sensor Platform^30^ by Spanish electronics manufacturer Libelium is an open physiological monitoring platform using nine different sensors: pulse, oxygen in blood (SpO_2_), airflow (breathing), body temperature, ECG, glucometer, galvanic skin response, blood pressure (sphygmomanometer) and patient position (accelerometer). The platform, shown in figures 3 and 4, consists of a board or shield that plugs into an Arduino or Raspberry Pi and interfaces with the various sensors, allowing for experimenting directly with the signals coming from these sensors.

To build the device a Raspberry Pi or Arduino is required. No special skills are required, as it comes as a modular kit where the various components are just plugged in. The components provided are off-the-shelf sensors and products that have been modified to work with the kit. Source code is available for Raspberry Pi and Arduino.

#### OpenBCI

**Figure 5:**
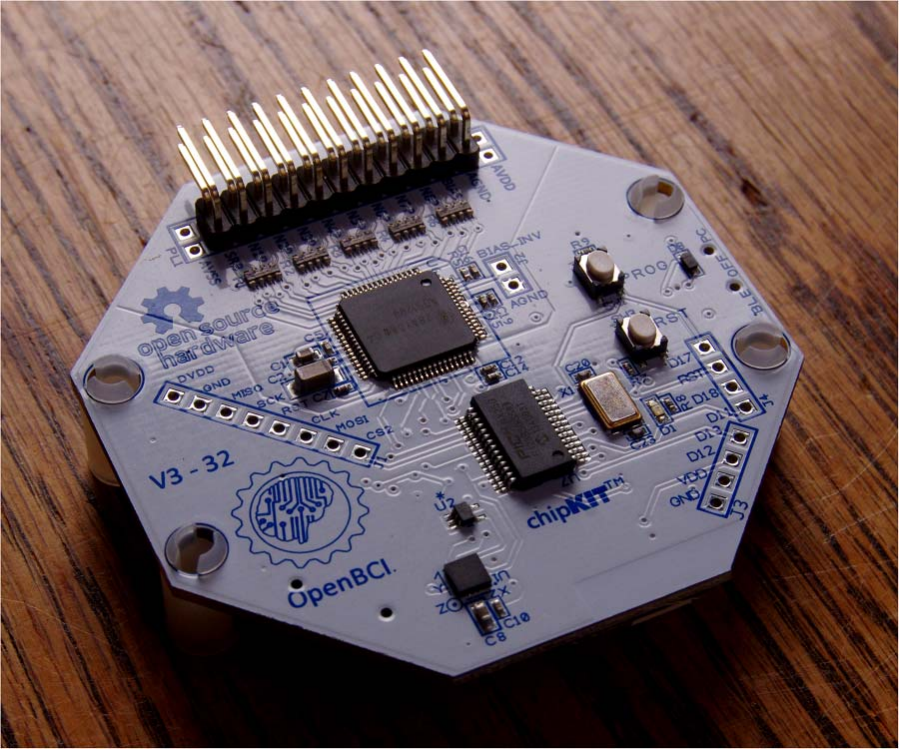
OpenBCI brain-computer interface (Photo by Wikimedia Commons, image licensed under the Creative Commons BY-SA license).

OpenBCI is an eight-channel EEG signal capture platform (see figure 5) of which the hardware, software and mechanical design files are available online.^31^ It is intended for human brain-computer interface technologies, but the hardware can also be used to perform other types of biosensing, like EMG and heart rate (EKG).

All hardware and electrodes are included with the kit. It comes as a modular kit, with the hardware design files available. A 3D-printable EEG headset, called Spiderclaw, is in development. Source code is available for Arduino (8-bit), ChipKit (32-bit), Processing and Python.

#### Do-it-yourself blood pressure monitor

Russell *et al*^32^ published instructions to build a do-it-yourself (DIY) blood pressure monitor. The instructions include a bill of materials and circuit diagrams. The blood pressure monitor relies on an electronic pressure sensor connected to an aneroid sphygmomanometer (a mechanical type with a dial). The monitor runs on batteries that can be recharged using a hand crank generator. They do mention that it is an experimental prototype and that the measurements should not be relied on for clinical use.

The device can be built using basic tools from a home hardware store. The various electronic components that can be sourced from distributors, as well as an off-the-shelf sphygmomanometer. An off-the-shelf project box is used to contain the electronics. Source code is available for a Microchip PIC microcontroller, and only basic knowledge of electronics and soldering is required.

### Diabetes

#### Diabeto

Diabeto is a small device that plugs into a glucometer and transfers blood glucose readings to a smartphone app. The data can then be displayed and analysed on the Diabeto web app, the mobile app as well as the Pebble smartwatch. The hardware design files, bill of materials and user instructions have been made available online.^33^

The Diabeto is available as a commercial off-the-shelf device, but the hardware design files are available online. The various electronic components can be sourced from distributors. The bill of materials includes manufacturer names and product codes. The enclosure and software do not seem to be open-source.

#### Nightscout xDrip

The Nightscout project^34^ is an open-source project that allows real-time access to a continuous glucose monitor (CGM) connected to a mobile phone, sending the data to a web app, smartphone app and smartwatch app. xDrip^35^ is an open-source hardware device that reads the wireless signals transmitted by the CGM sensor and sends data to a smartphone via Bluetooth Low-Energy (BLE). From the smartphone app the data can also be transmitted to the Nightscout database.

To build the device, a soldering iron and a Dexcom CGM is needed. There are four electronic components that can be sourced from open-source hardware distributors: BLE module, USB wireless module, battery and charger, and wires. Source code for an Android mobile app is available.

## Conclusions

Open-source medical devices are already available, even if they are not marketed as such. Table 1 shows a summary of the 10 devices that were compared in this paper. Evidently, open-source offers a solution to the high costs and slow pace of innovation of medical devices currently. Off-the-shelf healthcare sensors are available that can be connected to cheap embedded computers, and it is easy to construct practical systems that can be used for inventing new clinical applications that work. Rapidly achieving sophisticated results that would have been impossible only a year or two ago transforms one's attitude to medical devices: it is interesting, rewarding, and stimulates creative innovation.

**Table 1:** Summary of project comparison

Project	Estimated cost to build	OSHW license used	Microcontroller/platform used
MyOpen^10–13^	$250	GNU public license^10^	Blackfin DSP
e-Nable^16–18^	$350	Creative commons attribution non-commercial	Arduino
CT scanner^20–22^	$300	GNU public license V.3	Arduino
Fechko's peristaltic pump^26^	$170	N/A	Raspberry Pi or Arduino
Wijnen *et al*'s syringe pump^8^	$97^1^	Creative commons attribution share-alike	Raspberry Pi
e-Health sensor platform^30^	$500	Unknown	Raspberry Pi or Arduino
OpenBCI^31^	$450–$800	Creative commons share-alike	Arduino or ChipKit
DIY blood pressure monitor^32^	$50	N/A	Microchip PIC
Diabeto^33^	$50	Unknown	Atmel ATTiny
Nightscout xDrip^34^ ^35^	$60	N/A	Pololu Wixel

DIY, do-it-yourself; DSP, digital signal processing; N/A, not applicable; OSHW, open-source hardware.

There remain many challenges for open-source hardware in healthcare. Medical devices need to be regulated, and this can be time consuming and expensive, but still quite manageable for an open-source project. Crowdfunding is one way of getting the funds together to apply for certification. There are successful open business models for open-source hardware, making it possible to invest in them as commercial entities. Finally, note that while open-source hardware does not use patents for intellectual property protection, open-source hardware companies generally do use trademarks to protect their brands and to assure a level of quality in the products.
